# Effects of supplemental octanoate on hepatic lipid metabolism, serum biochemical indexes, antioxidant capacity and inflammation-related genes expression of large yellow croaker (*Larimichthys crocea*) fed with high soybean oil diet

**DOI:** 10.3389/fimmu.2023.1162633

**Published:** 2023-03-27

**Authors:** Manxi Zhao, Zhou Zhang, Yongtao Liu, Wencong Zhang, Ye Gong, Yuhang Tang, Fan Chen, Jianmin Zhang, Guobin Liu, Haitao Zhang, Yueru Li, Kangsen Mai, Qinghui Ai

**Affiliations:** ^1^ Key Laboratory of Aquaculture Nutrition and Feed (Ministry of Agriculture and Rural Affairs) and Key Laboratory of Mariculture (Ministry of Education), Ocean University of China, Qingdao, China; ^2^ Guangdong Evergreen Feed Industry Co., Ltd., Key Laboratory of Aquatic, Livestock and Poultry Feed Science and Technology in South China, Ministry of Agriculture and Rural Affairs, Zhanjiang, China; ^3^ Laboratory for Marine Fisheries Science and Food Production Processes, Qingdao National Laboratory for Marine Science and Technology, Qingdao, China

**Keywords:** octanoate, large yellow croaker, lipid metabolism, antioxidant capacity, inflammatory response

## Abstract

Dietary high soybean oil (SO) levels might cause hepatic lipid deposition, induce oxidative stress and inflammatory response in aquatic animals, while octanoate (OCT) is beneficial to metabolism and health in mammals. However, the effect of OCT has been studied rarely in aquatic animals. In this study, a 10-week feeding trial was conducted to investigate the effect of supplemental OCT on hepatic lipid metabolism, serum biochemical indexes, antioxidant capacity and inflammatory response of large yellow croaker (*Larimichthys crocea*) fed with high SO levels diet. The negative control diet contained 7% fish oil (FO), while the positive control diet contained 7% SO. The other four experimental diets were supplemented with 0.7, 2.1, 6.3 and 18.9 g/kg sodium octanoate (OCT) based on the positive control diet. Results showed that OCT supplementation effectively reduced the hepatic crude lipid, triglyceride (TG), total cholesterol (TC) and non-esterified free fatty acids contents, and alleviated lipid accumulation caused by the SO diet. Meanwhile, OCT supplementation decreased the serum TG, TC, alanine transaminase, aspartate transaminase and low-density lipoprotein cholesterol levels, increased the serum high-density lipoprotein cholesterol level, improved the serum lipid profiles and alleviated hepatic injury. Furthermore, with the supplementation of OCT, the mRNA expression of genes related to lipogenesis (*acc1*, *scd1*, *fas*, *srebp1*, *dgat1* and *cebpα*) and fatty acid (FA) transport (*fabp3, fatp* and *cd36*) were down-regulated, while the mRNA expression of genes related to lipolysis (*atgl*, *hsl* and *lpl*) and FA β-oxidation (*cpt1* and *mcad*) were up-regulated. Besides that, dietary OCT increased the total antioxidant capacity, activities of peroxidase, catalase and superoxide dismutase and the content of reduced glutathione, decreased the content of 8-hydroxy-deoxyguanosine and malondialdehyde and relieved hepatic oxidative stress. Supplementation of 0.7 and 2.1 g/kg OCT down-regulated the mRNA expression of genes related to pro-inflammatory cytokines (*tnfα*, *il1β* and *ifnγ*), and suppressed hepatic inflammatory response. In conclusion, supplementation with 0.7-2.1 g/kg OCT could reduce hepatic lipid accumulation, relieve oxidative stress and regulate inflammatory response in large yellow croaker fed the diet with high SO levels, providing a new way to alleviate the hepatic fat deposition in aquatic animals.

## 1 Introduction

Soybean oil (SO) enriched in n-6 polyunsaturated fatty acids (n-6 PUFA) has been applied to the aquaculture industry widely, because of its high output and low price. A previous study found 8% SO in diet could alter the fatty acid composition of juvenile hybrid sturgeon (*Acipenser Baeribrandt♀×A. schrenckiibrandt♂*) (1). Similar results were found in grass carp (*Ctenopharyngodon Idella*) fed diets with 5% SO diet (2) and largemouth bass (*Micropterus Salmoides*) fed diets with 9% SO (3). Supplementation of 56% and 100% SO in the diet (8% dietary lipid) significantly increased hepatic lipid content in blunt snout bream (*Megalobrama Amblycephala*) juvenile (4). And 7.5% SO in diet increased lipid deposition in the liver of turbot (*Scophthalmus Maximus L.*) (5). Meanwhile, dietary 6.5% SO depressed the hepatic anti-oxidative capacity and induced the inflammatory response in large yellow croaker (*Larimichthys crocea*) (6). Therefore, it is necessary to find suitable additives to alleviate metabolic abnormalities caused by high levels of SO.

Octanoate (OCT) is a medium-chain fatty acid (MCFA) that is found in goat’s milk and coconut oil (7). Studies showed that dietary OCT could inhibit fat accumulation (8), ameliorate insulin resistance (9), suppress oxidative stress (10) and inflammatory response (11) in mammals. In aquatic animals, studies showed that OCT could affect the FA perception of rainbow trout (*Oncorhynchus mykiss*) (12), and improve oxidative stress in the gut-brain axis of zebrafish (*Danio rerio*) (13). However, there are few studies on effects of OCT on hepatic lipid metabolism, antioxidant capacity and inflammatory response in fish fed with high SO levels.

Large yellow croaker is an important economic fish in mariculture and a good model for studying fish lipid metabolism (14). At present, studies found that high-level SO diets could induce fat deposition, oxidative stress and inflammatory response of large yellow croaker (15–17). Considering the beneficial effect of OCT on metabolism and health in mammals, we investigated the effects of supplemental OCT on lipid metabolism, antioxidant capacity and inflammatory response of large yellow croaker fed the diet with high SO level. Our findings can be used to investigate effective nutritional strategies to alleviate fat deposition in aquatic animals.

## 2 Materials and methods

### 2.1 Experimental procedure

Large yellow croaker juveniles were provided by FuFa Aquatic Products Co., Ltd. (Ningde, China). The preparation of experimental diets referred to the previous study (18) (**Supplementary Table 1**). Diets containing the negative control group with 7% fish oil (FO) as the lipid source, the positive control group with 7% SO as the lipid source and treatment diets (to the SO group, 0.7, 2.1, 6.3 and 18.9 g/kg sodium octanoate (OCT) (purity: ≥ 99%, Sigma, USA) were added). Before the formal experiment, all the fish were fed commercial diets for 2 weeks to acclimate to experimental conditions. Fish were fasted for 24 h prior to the start of the feeding trial. Then 60 fish of similar size (13.00 ± 0.10 g) were randomly divided into 18 cages (1 m × 1 m × 1.5 m). Each diet was fed randomly to 3 cages twice a day (05:00 and 17:00) for 10 weeks in suitable environmental conditions (temperature: 19.5-25.5°C, salinity: 25-28‰, and dissolved oxygen content was approximately 7 mg/L).

### 2.2 Sample collection

At the end of the feeding trial, fish were starved for 24 h, then anesthetized using MS-222 (1: 10,000, Sigma) for sample collection. Blood samples were collected from the caudal vein to anticoagulant-free centrifuge tubes, clotted at 4°C for 4 h, then centrifuged at 2, 500 ×g (10 min) to separate serum for biochemical index analysis. Hepatic samples from five fish per cage were fixed in 4% paraformaldehyde for 24 h, then transferred to 75% ethanol for hematoxylin and eosin (H&E) staining and the Oil Red O staining. Hepatic tissues were collected from ten fish per cage, frozen in liquid nitrogen, and stored at −80°C for subsequent analysis.

### 2.3 Hepatic histological analysis

Paraffin-embedded sections and frozen sections of hepatic tissue were made according to the method described in a published paper (19). The paraffin sections were stained with hematoxylin and eosin and the frozen sections were stained with Oil Red O. Images were captured and analyzed using the microscope (Olympus BX53, Japan). The results of the Oil Red O area were analyzed using Image Pro Plus 6.0 software (Media Cybernetics, USA).

### 2.4 Hepatic biochemical analysis

The moisture and crude lipid of hepatic tissues were measured according to previous methods (19). Moisture was measured by drying samples at 105°C ventilation drying oven to constant weight. The crude lipid was measured by petroleum ether extraction using Soxhlet (Buchi, Sweden) to constant weight. Hepatic triglyceride (TG), total cholesterol (TC) and non-esterified fatty acids (NEFA) contents were detected by using kits (Nanjing Jiancheng Bioengineering Institute, China).

### 2.5 Serum biochemical index analysis

Serum TG, TC, high-density lipoprotein cholesterol (HDL-c), low-density lipoprotein cholesterol (LDL-c) contents and activities of alkaline phosphatase (ALP), aspartate transaminase (AST) and alanine transaminase (ALT) were measured by using kits (Nanjing Jiancheng Bioengineering Institute, China).

### 2.6 Hepatic fatty acids composition analysis

The FAs profiles of hepatic tissues were measured with Gas Chromatograph-Mass Spectrometer (GC-MS, QP2010 Plus, SHIMADZU, Japan) according to the method described by a previous study (20) with some modifications. The samples were subjected to a series of pre-treatments and finally quantified by GC-MS.

### 2.7 Hepatic antioxidant capacity analysis

Accurately weighed the weight of the hepatic tissue, added 9 times of normal saline to make the tissue homogenate, centrifuged at 2,500 rpm (10 min), then the supernatant was used for testing. Protein concentration was determined by a BCA Protein Assay Kit (Beyotime Institute of Technology, China). The total antioxidant capacity (T-AOC), activities of peroxidase (POD), catalase (CAT), superoxide dismutase (SOD) and contents of reduced glutathione (GSH), malondialdehyde (MDA), 8-hydroxydeoxyguanosine (8-OHdG) of liver were measured by using kits (Nanjing Jiancheng Bioengineering Institute, China).

### 2.8 RNA extraction, cDNA synthesis and real-time quantitative PCR (RT-qPCR) analysis

The RNA extraction followed the method of the previous study (20). Then, RNA was reversely transcribed to cDNA using the Prime Script-RT reagent Kit (Vazyme, China). RT-qPCR was carried out on a CFX96 Real-Time PCR Detection System (BIO-RAD, USA). The amplification was performed in a total volume of 20 μL containing 4 μL of cDNA, 0.5 μL of each primer, 10 μL of ChamQ Universal SYBR qPCR Master Mix (Vazyme, China) and 5 μL of RNase-free water. Real-time quantitative PCR procedures were 95°C for 2 min, followed by 39 cycles of 95°C for 10 s, 58°C for 10 s, and 72°C for 20 s. The primers used for RT-qPCR were presented in **Table 1**. Relative gene expression levels were calculated with the 2^–ΔΔCT^ method (14).

**Table 1:** Primers used for real-time quantitative PCR.

Primers	Forward(5’-3’)	Reverse(5’-3’)	Reference
*acc1*	GACTTGGCGGAATACCTACTGG	GCTTGCTGGATGATCTTTGCTT	(21)
*scd1*	AAAGGACGCAAGCTGGAACT	CTGGGACGAAGTACGACACC	(22)
*fas*	CAGCCACAGTGAGGTCATCC	TGAGGACATTGAGCCAGACAC	(23)
*srebp1*	TCTCCTTGCAGTCTGAGCCAAC	TCAGCCCTTGGATATGAGCCT	(24)
*dgat1*	GGTATCTTGGTGGACCCCATTCA	TGAGCACCGTGGCTGAAGGAAAGA	(22)
*cebpα*	GAGGCGGGAAGCACAAGAAG	TTCGCCTTGTCGCGGCTCTTAC	(22)
*cd36*	GAGCATGATGGAAAATGGTTCAAAG	CTCCAGAAACTCCCTTTCACCTTAG	(23)
*fatp1*	CAACCAGCAGGACCCATTACG	CATCCATCACCAGCACATCACC	(23)
*apob100*	AGAGTGTTGTCCAGGATAAAGATGC	CAGGGCTCAGGGTCTCAGTC	(21)
*fabp3*	CCAAACCCACCACTATCATCTCAG	GCACCATCTTTCCCTCCTCTATTG	(23)
*atgl*	CCATGCATCCGTCCTTCAACC	GAGATCCCTAACCGCCCACT	(23)
*hsl*	TCTCTCTGTCGCTGGGGC	GGGACTTGGAGGTTTGGG	(23)
*lpl*	GAGAGGATTCATCTGCTGGGTTAC	ACATCAACAAACTGGGCGTCATC	(24)
*pparα*	GTCAAGCAGATCCACGAAGCC	TGGTCTTTCCAGTGAGTATGAGCC	(25)
*cpt1*	GCTGAGCCTGGTGAAGATGTTC	TCCATTTGGTTGAATTGTTTACTGTCC	(23)
*aco*	AGTGCCCAGATGATCTTGAAGC	CTGCCAGAGGTAACCATTTCCT	(23)
*mcad*	GCTGAGATGGCAATGAAGGTGGAG	GATGGAGGCGTAGTAGGTGTTTCTG	(26)
*il10*	AGTCGGTTACTTTCTGTGGTG	TGTATGACGCAATATGGTCTG	(27)
*tnfα*	ACACCTCTCAGCCACAGGAT	CCGTGTCCCACTCCATAGTT	(27)
*il1β*	CATAGGGATGGGGACAACGA	AGGGGACGGACACAAGGGTA	(27)
*ifnγ*	TCAGACCTCCGCACCATCA	GCAACCATTGTAACGCCACTTA	(27)
*β-actin*	CTACGAGGGTTATGCCCTGCC	TGAAGGAGTAACCGCGCTCTGT	(23)

*acc1*, acetyl CoA carboxylase 1; *scd1*, stearoyl-coenzyme A desaturase 1; *fas*, fatty acid synthase; *srebp1*, sterol-regulatory element binding protein-1; *dgat1*, diacylglycerol acyltransferase 1; *cebpα*, CCAAT/enhancer binding protein α; *cd36*, cluster of differentiation 36; *fatp1*, fatty acid transport protein 1; *apob100*, apolipoprotein B 100; *fabp3*, fatty acid binding protein 3; *atgl*, adipose triglyceride lipase; *hsl*, hormone-sensitive triglyceride lipase; *lpl*, lipoprotein lipase; *pparα*, peroxisome proliferator-activated receptor α; *cpt1*, carnitine palmitoyl transferase 1; *aco*, acyl-CoA oxidase; *mcad*, medium chain acyl-CoA dehydrogenase; *il10*, interleukin-10; *tnfα*, tumor necrosis factor-α; *il1β*, interleukin-1β; *ifnγ*, interferon-γ.

### 2.9 Statistical analysis

All statistics were performed by SPSS 23.0 (IBM, America). The normality and homogeneity of variances were examined first. Then, two hypotheses were used in this study: (1) Independent-samples T-test was used in fish fed the FO diet and the SO diet. (2) A one-way analysis of variance (ANOVA) followed by Tukey’s multiple-range test and polynomial contrasts analysis was used in fish fed the diet with SO and OCT (0.7, 2.1, 6.3, 18.9 g/kg). The significance threshold was set at *P* < 0.05. The results were presented as means ± S.E.M. (standard error of the mean).

## 3 Results

### 3.1 Hepatic morphology

**Figure 1:**
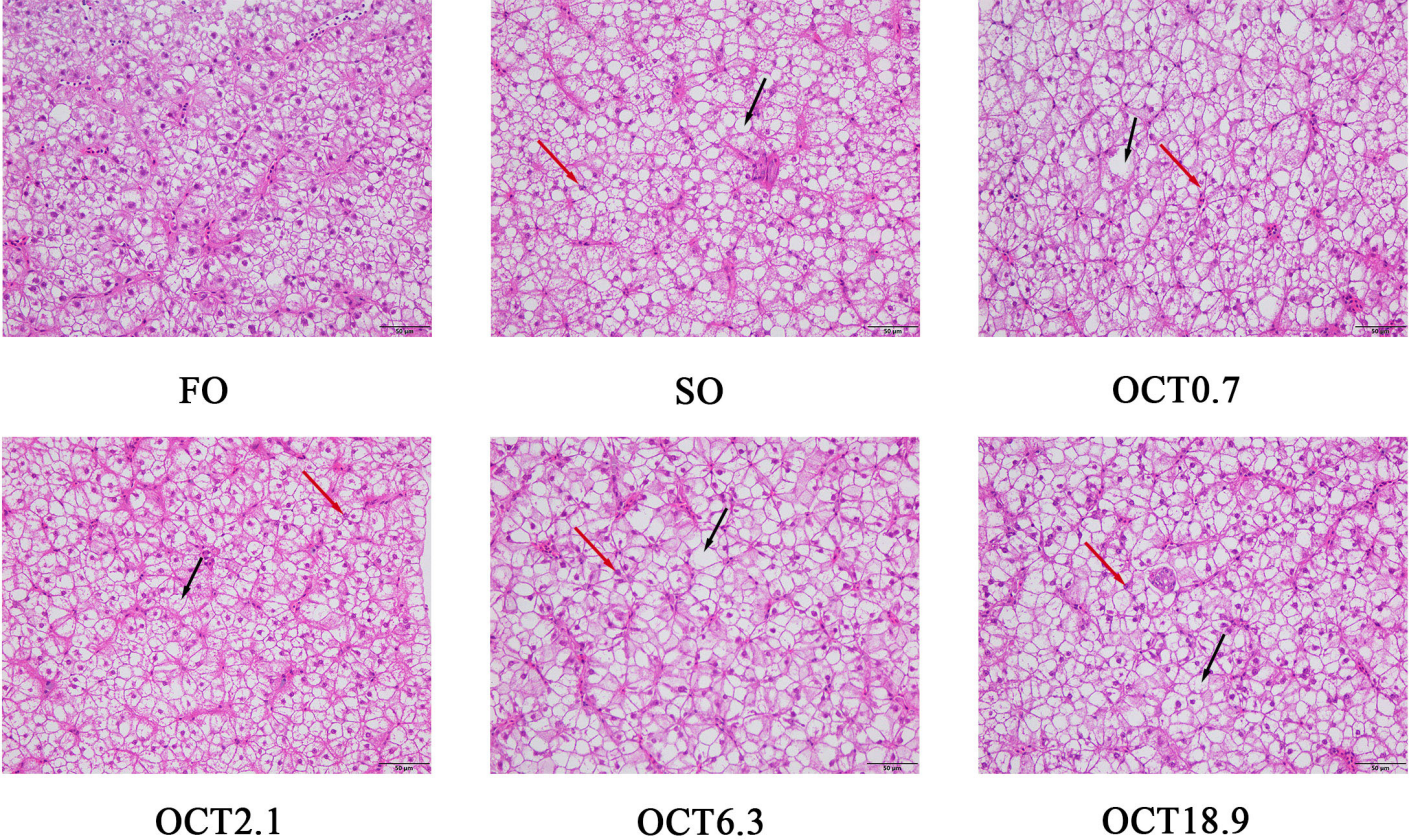
Effects of supplemental octanoate on hepatic morphology of large yellow croaker. The H&E staining results of different dietary treatments (200×, bars = 50 µm). The black arrows point to cellular vacuoles and the red arrows point to nuclear migration.

The hepatic morphology was performed by H&E staining. Compared with the FO diet, hepatocytes showed significant vacuolation and nuclear migration in fish fed with the SO diet (**Figure 1**). The supplementation of OCT reduced the size and number of vacuoles and alleviated nuclear atrophy and deviation, especially in fish fed the diet containing 2.1 g/kg OCT.

### 3.2 Hepatic biochemical index and Oil red O staining

**Figure 2:**
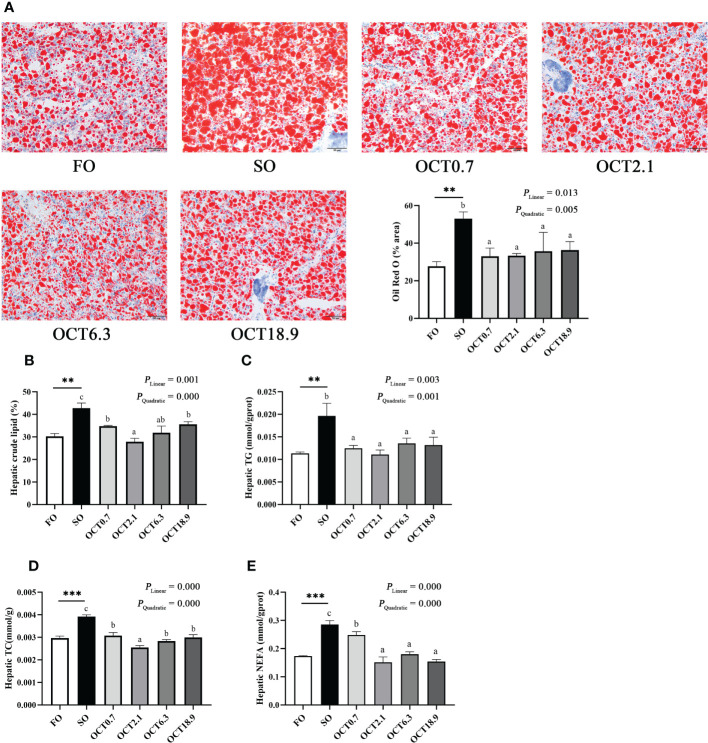
Effects of supplemental octanoate on hepatic lipid deposition of large yellow croaker. **(A)** The oil red O staining results of different dietary treatments (200×, bars = 50 µm), **(B)** Lipid contents in liver, **(C)** Total triglyceride contents in liver, **(D)** Total cholesterol contents in liver, **(E)** The non-esterified fatty acids contents in liver. The values were means (n = 3) with their standard errors represented by vertical bars. and the FO group. ∗∗ represent a significant difference of *P* < 0.01 between the SO group and the FO group. ∗∗∗ represent a significant difference of *P* < 0.001 between the SO group and the FO group. Different letters show significant difference (*P* < 0.05) among OCT treatment groups. Linear trend and Quadratic trend were analyzed by orthogonal polynomial contrasts.

The liver is an important metabolic organ involved in lipid metabolism. The results of Oil red O staining showed that the area of lipid droplets (LDs) was significantly increased when the FO in the diet was completely replaced by SO (*P* < 0.05). In contrast, OCT supplementation decreased the area of LDs in significantly linear and quadratic patterns (*P* < 0.05) (**Figure 2A**). Meanwhile, hepatic crude lipid, TG, TC and NEFA contents in fish fed the SO diet were notably higher than those in the FO diet (*P* < 0.01) and were effectively reduced by OCT supplementation (**Figures 2B–E**). As the OCT level increased, these were decreased in significantly linear and quadratic patterns (*P* < 0.05), and the peak values were observed in fish fed the diet containing 2.1 g/kg OCT.

### 3.3 Hepatic fatty acids composition

Hepatic FAs composition analysis (**Table 2**) showed that total saturated fatty acids (SFA) and total n-3 polyunsaturated fatty acids (n-3 PUFA) were significantly decreased and total n-6 PUFA were significantly increased when the FO in the diet was completely replaced by SO (*P* < 0.05). With the addition of OCT, the relative content of C18:2n-6 and C20:4n-6 were decreased in a significantly linear pattern (*P* < 0.05). The relative content of C22:6n-3 and the ratio of n-3 PUFA to n-6 PUFA showed a significant quadratic curve (*P* < 0.05) and maximum were observed in fish fed the diet containing 0.7 g/kg OCT.

**Table 2:** Fatty acid profiles in liver of large yellow croaker in different groups (% total fatty acids).

Fatty acid (%)	Diets						Polynomial contrasts		
	FO	SO	OCT0.7	OCT2.1	OCT6.3	OCT18.9	*P*-value	Linear	Quadratic
C14:0	5.19 ± 0.50	1.42 ± 0.03^**^	1.42 ± 0.03	1.73 ± 0.03	1.40 ± 0.12	1.93 ± 0.64	0.623	0.297	0.712
C16:0	34.50 ± 0.14	24.66 ± 0.08^***^	27.08 ± 0.05	26.05 ± 0.29	28.89 ± 2.13	29.46 ± 2.84	0.260	0.047	0.979
C18:0	6.45 ± 0.36	6.30 ± 0.23^a^	7.06 ± 0.21^a^	7.92 ± 0.12^a^	10.96 ± 0.93^b^	10.85 ± 0.68^b^	0.000	0.000	0.831
ΣSFA^1^	46.15 ± 0.81	32.38 ± 0.28^***^a^^	35.55 ± 0.17^ab^	35.71 ± 0.29^ab^	41.26 ± 1.71^b^	42.25 ± 2.89^b^	0.004	0.000	0.860
C16:1n-7	6.23 ± 0.06	2.96 ± 0.07^***^b^^	2.53 ± 0.07^ab^	2.85 ± 0.06^b^	1.94 ± 0.13^a^	2.66 ± 0.34^ab^	0.014	0.052	0.131
C18:1n-9	16.05 ± 0.21	18.58 ± 0.32^**^	17.90 ± 0.30	18.02 ± 0.10	16.10 ± 1.53	17.32 ± 0.92	0.334	0.129	0.585
ΣMUFA^1^	22.28 ± 0.26	21.53 ± 0.39	20.43 ± 0.23	20.87 ± 0.09	18.04 ± 1.63	19.97 ± 0.62	0.093	0.056	0.377
C18:2n-6	7.73 ± 0.50	25.90 ± 0.88^***^b^^	18.74 ± 1.03^a^	19.29 ± 0.73^a^	20.18 ± 1.71^a^	17.39 ± 1.29^a^	0.004	0.002	0.067
C20:4n-6	0.16 ± 0.01	0.04 ± 0.00^**^c^^	0.04 ± 0.01^c^	0.03 ± 0.00^bc^	0.02 ± 0.00^ab^	0.01 ± 0.00^a^	0.001	0.000	0.251
Σn-6 PUFA^1^	7.90 ± 0.50	25.94 ± 0.88^***b^	18.78 ± 1.02^a^	19.32 ± 0.73^a^	20.20 ± 1.72^a^	17.40 ± 1.29^a^	0.004	0.002	0.068
C18:3n-3	1.09 ± 0.06	2.00 ± 0.07^**b^	1.91 ± 0.10^ab^	1.88 ± 0.04^ab^	1.57 ± 0.27^ab^	1.26 ± 0.11^a^	0.023	0.002	0.201
C20:5n-3	1.63 ± 0.04	0.32 ± 0.00^***^	0.34 ± 0.03	0.31 ± 0.02	0.31 ± 0.02	0.29 ± 0.03	0.607	0.238	0.428
C22:6n-3	2.88 ± 0.05	0.49 ± 0.02^***ab^	0.69 ± 0.03^c^	0.52 ± 0.03^b^	0.41 ± 0.04^ab^	0.37 ± 0.02^a^	0.000	0.000	0.003
Σn-3 PUFA^1^	5.61 ± 0.14	2.81 ± 0.04^***bc^	2.96 ± 0.07^c^	2.71 ± 0.04^bc^	2.29 ± 0.25^ab^	1.91 ± 0.15^a^	0.002	0.000	0.036
n-3/n-6 PUFA	0.71 ± 0.03	0.11 ± 0.00^***a^	0.16 ± 0.01^b^	0.14 ± 0.00^ab^	0.11 ± 0.00^a^	0.11 ± 0.02^a^	0.003	0.172	0.002
Σn-3 LC-PUFA^1^	4.52 ± 0.09	0.81 ± 0.03^***ab^	1.04 ± 0.04^c^	0.83 ± 0.05^b^	0.72 ± 0.02^ab^	0.65 ± 0.04^a^	0.000	0.000	0.005

^a^Data are means of triplicate and presented as mean ± SEM. ** represent a significant difference of *P* < 0.01 between the SO group and the FO group. *** represent a significant difference of *P* < 0.001 between the SO group and the FO group. Different letters show significant difference (*P* < 0.05) among OCT treatment groups. Linear trend and Quadratic trend were analyzed by orthogonal polynomial contrasts.

^1^SFA, saturated fatty acids; MUFA, mono-unsaturated fatty acids; n-6 PUFA, n-6 polyunsaturated fatty acids; n-3 PUFA, n-3 polyunsaturated fatty acid; n-3 LC-PUFA, n-3 long chain polyunsaturated fatty acids.

### 3.4 Serum biochemical index

**Figure 3:**
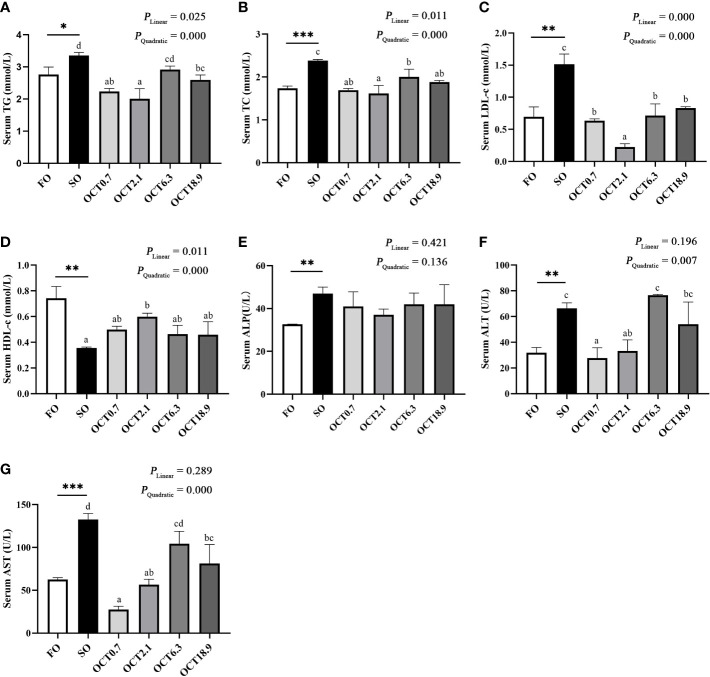
Effects of supplemental octanoate on serum biochemical indexes of large yellow croaker. **(A)** Serum TG contents, **(B)** Serum TC contents, **(C)** Serum LDL-c contents, **(D)** Serum HDL-c contents, **(E)** Serum ALP activity, **(F)** Serum ALT activity, **(G)** Serum AST activity. Values were means (n = 3) with their standard errors represented by vertical bars. ∗ represent a significant difference of *P* < 0.05 between the SO group and the FO group. ∗∗ represent a significant difference of *P* < 0.01 between the SO group and the FO group. ∗∗∗ represent a significant difference of *P* < 0.001 between the SO group and the FO group. Different letters show significant difference (*P* < 0.05) among OCT treatment groups. Linear trend and Quadratic trend were analyzed by orthogonal polynomial contrasts.

The serum TG, TC and LDL-c levels were significantly increased when the FO in the diet was completely replaced by SO (*P* < 0.05) (**Figures 3A–C**). In contrast, OCT supplementation decreased these in a significantly quadratic pattern (*P* < 0.001), and peak values were observed in fish fed the diet containing 2.1 g/kg OCT. The serum HDL-c level was increased in a significantly quadratic pattern with increasing dietary OCT (*P* < 0.01) (**Figure 3D**). The serum ALP levels were not significantly affected by the supplementation of OCT (*P* > 0.05) (**Figure 3E**). Both the serum ALT and AST levels were decreased in a significantly quadratic pattern with the increase of dietary OCT (*P* < 0.05) (**Figures 3F, G**), and the values in fish fed diets containing 0.7 and 2.1 g/kg OCT were lower.

### 3.5 The mRNA expression of genes related to lipid metabolism in liver

**Figure 4:**
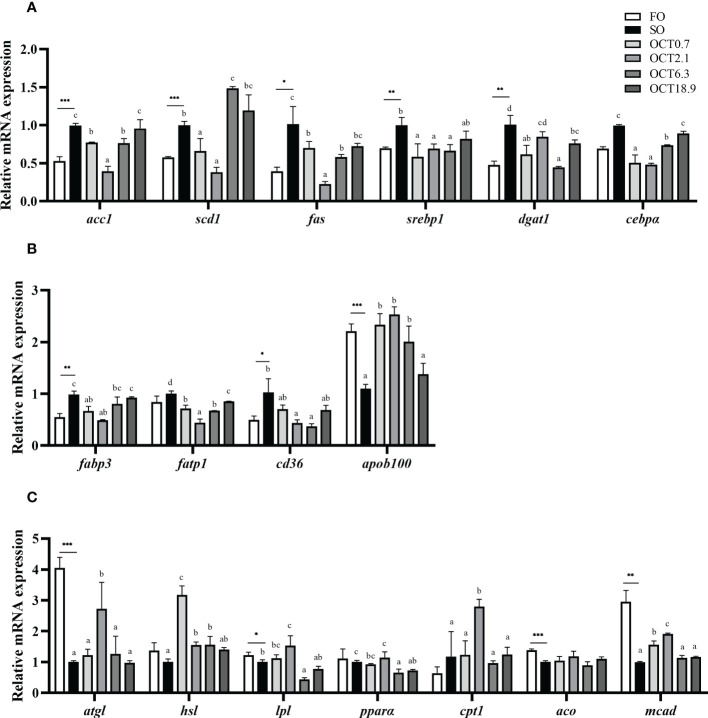
Effects of supplemental octanoate on the expression of lipid metabolism-related genes in the liver of large yellow croaker. **(A)** Lipogenesis, **(B)** FA transport, **(C)** Lipolysis and FA oxidation. Values were means (n = 3) with their standard errors represented by vertical bars. ∗ represent a significant difference of *P* < 0.05 between the SO group and the FO group. ∗∗ represent a significant difference of *P* < 0.01 between the SO group and the FO group. ∗∗∗ represent a significant difference of *P* < 0.001 between the SO group and the FO group. Different letters show significant difference (*P* < 0.05) among OCT treatment groups. *acc1*, acetyl CoA carboxylase 1; *scd1*, stearoyl-coenzyme A desaturase 1; *fas*, fatty acid synthase; *srebp1*, sterol-regulatory element binding protein-1; *dgat1*, diacylglycerol acyltransferase 1; *cebpα*, CCAAT/enhancer binding protein α; *fabp3*, fatty acid binding protein 3; *fatp1*, fatty acid transport protein 1; *cd36*, cluster of differentiation 36; *apob100*, apolipoprotein B 100; *atgl*, adipose triglyceride lipase; *hsl*, hormone-sensitive triglyceride lipase; *pparα*, peroxisome proliferator-activated receptor α; *cpt1*, carnitine palmitoyl transferase 1; *aco*, acyl-CoA oxidase; *mcad*, medium chain acyl-CoA dehydrogenase. Polynomial analysis in **(A)**: *acc1*: *P*_Linear_ = 0.479, *P*_Quadratic_ = 0.000; *scd1*: *P*_Linear_ = 0.000, *P*_Quadratic_ = 0.000; *fas*: *P*_Linear_ = 0.006, *P*_Quadratic_ = 0.000; *srebp1*: *P*_Linear_ = 0.194, *P*_Quadratic_ = 0.002; *dgat1*: *P*_Linear_ = 0.001, *P*_Quadratic_ = 0.002; *cebpα*: *P*_Linear_ = 0.856, *P*_Quadratic_ = 0.000. Polynomial analysis in **(B)**: *fabp3*: *P*_Linear_ = 0.926, *P*_Quadratic_ = 0.000; *fatp1*: *P*_Linear_ = 0.003, *P*_Quadratic_ = 0.000; *cd36*: *P*_Linear_ = 0.002, *P*_Quadratic_ = 0.001; *apob100*: *P*_Linear_ = 0.539, *P*_Quadratic_ = 0.000. Polynomial analysis in **(C)**: *atgl*: *P*_Linear_ = 0.985, *P*_Quadratic_ = 0.003; *hsl*: *P*_Linear_ = 0.044, *P*_Quadratic_ = 0.000; *lpl*: *P*_Linear_ = 0.003, *P*_Quadratic_ = 0.013; *pparα*: *P*_Linear_ = 0.001, *P*_Quadratic_ = 0.088; *cpt1*: *P*_Linear_ = 0.878, *P*_Quadratic_ = 0.012; *aco*: *P*_Linear_ = 0.829, *P*_Quadratic_ = 0.735; *mcad*: *P*_Linear_ = 0.460, *P*_Quadratic_ = 0.000. Linear trend and Quadratic trend were analyzed by orthogonal polynomial contrasts.

Compared with the FO diet, the mRNA expression of genes related to lipogenesis including *acc1*, *scd1*, *fas*, *srebp1*, *dgat1* and *cebpα* were significantly up-regulated in fish fed the SO diet (*P* < 0.05) (**Figure 4A**). With the dietary OCT increasing, the mRNA expression of these genes showed a significant quadratic curve (*P* < 0.05), and the values in fish fed diets containing 0.7 and 2.1 g/kg OCT were lower than the SO diet. The mRNA expression of FA transport-related genes, such as *fabp3* and *cd36*, were significantly up-regulated and *apob100* was significantly down-regulated when the FO in the diet was completely replaced by SO (*P* < 0.05) (**Figure 4B**). With the OCT level increasing, the mRNA expression of *fabp3, fatp1* and *cd36* were down-regulated in a significantly quadratic pattern, while the mRNA expression of *apob100* showed the opposite trend (*P* < 0.05).

Compared with the FO diet, the mRNA expression of genes related to lipolysis, such as *atgl* and *lpl*, were significantly down-regulated in fish fed the SO diet (*P* < 0.05) (**Figure 4C**). With OCT level increasing, the mRNA expression of *atgl* and *hsl* showed a significant quadratic curve (*P* < 0.05), and maximum were observed in fish fed the diet containing 2.1 g/kg OCT and 0.7 g/kg OCT. The mRNA expression of genes related to FA β-oxidation including *aco* and *mcad* were significantly lower, while the mRNA expression of *cpt1* was significantly higher in fish fed the SO diet than those in the FO diet (*P* < 0.05). With OCT level increasing, the mRNA expression of *cpt1* and *mcad* showed a significant quadratic curve (*P* < 0.05), and maximum were observed in fish fed the diet containing 2.1 g/kg OCT.

### 3.6 Hepatic antioxidant activities

**Figure 5:**
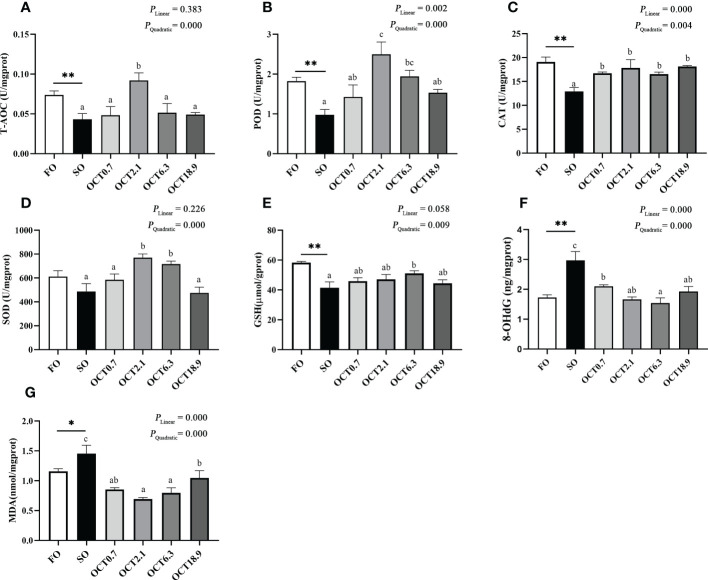
Effects of supplemental octanoate on hepatic antioxidant capacity of large yellow croaker. **(A)** Total antioxidant capacity, **(B)** Peroxidase activity, **(C)** Catalase activity, **(D)** Superoxide dismutase activity, **(E)** Reduced glutathione contents, **(F)** 8-OHdG contents, **(G)** Malondialdehyde contents. Values were means (n = 3) with their standard errors represented by vertical bars. ∗ represent a significant difference of *P* < 0.05 between the SO group and the FO group. ∗∗ represent a significant difference of *P* < 0.01 between the SO group and the FO group. Different letters show significant difference (*P* < 0.05) among OCT treatment groups. Linear trend and Quadratic trend were analyzed by orthogonal polynomial contrasts.

The T-AOC and activities of POD and CAT were significantly decreased when the FO in the diet was completely replaced by SO (*P* < 0.05) (**Figures 5A–C**). With the increase of dietary OCT, the T-AOC and activities of POD, CAT and SOD showed a significant quadratic curve (*P* < 0.05), and maximum were observed in fish fed the diet containing 2.1 g/kg OCT (**Figures 5A–D**). Compared with the FO diet, the content of GSH, 8-OHdG and MDA were significantly increased in fish fed the SO diet (*P* < 0.05) (**Figures 5E–G**). As the OCT level increased, the content of GSH was increased in a significantly quadratic pattern (*P* < 0.05), while the contents of 8-OHdG and MDA were decreased in significantly linear and quadratic patterns (*P* < 0.05).

### 3.7 The mRNA expression of genes related to inflammation in liver

**Figure 6:**
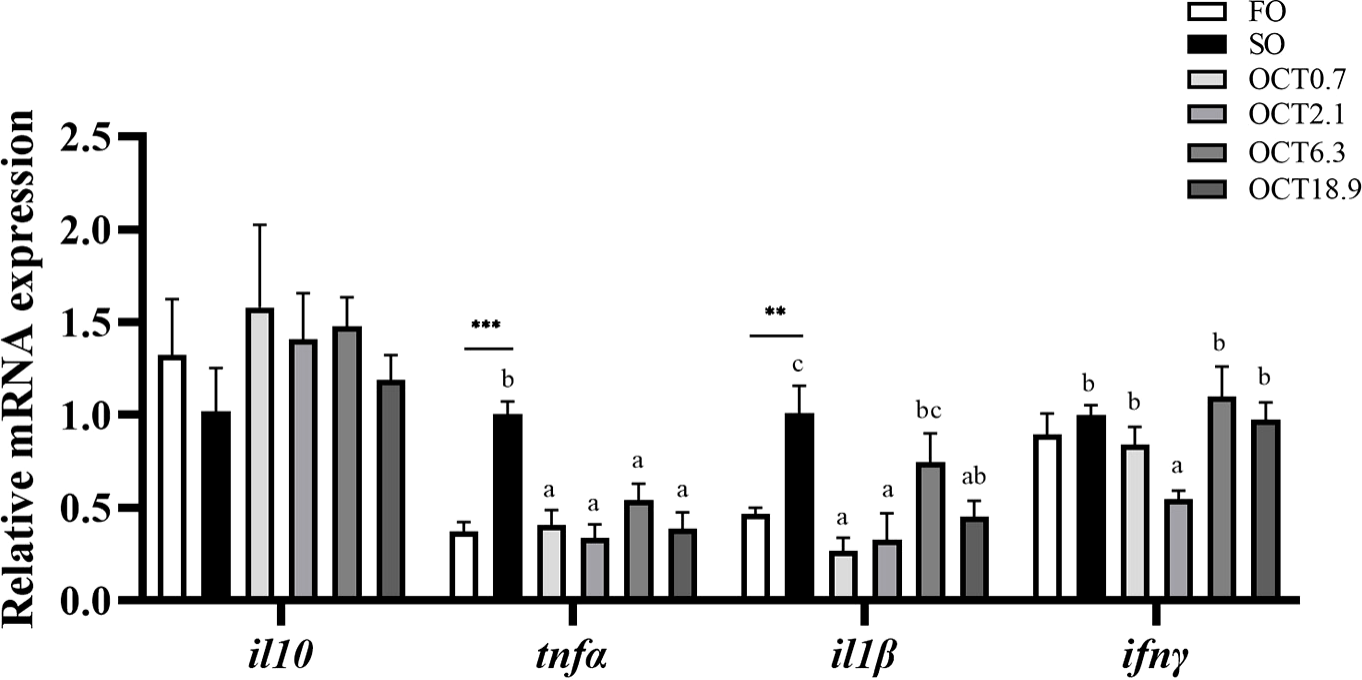
Effects of supplemental octanoate on the expression of inflammation-related genes in the liver of large yellow croaker. Values were means (n = 3) with their standard errors represented by vertical bars. ∗∗ represent a significant difference of *P* < 0.01 between the SO group and the FO group. ∗∗∗ represent a significant difference of *P* < 0.001 between the SO group and the FO group. Different letters show significant difference (*P* < 0.05) among OCT treatment groups. Linear trend and Quadratic trend were analyzed by orthogonal polynomial contrasts. *il10*, interleukin-10; *tnfα*, tumor necrosis factor-α; *il1β*, interleukin-1β; *ifnγ*, interferon-γ. Polynomial analysis: *il10*: *P*_Linear_ = 0.638, *P*_Quadratic_ = 0.031; *tnfα*: *P*_Linear_ = 0.000, *P*_Quadratic_ = 0.000; *il1β*: *P*_Linear_ = 0.019, *P*_Quadratic_ = 0.001; *ifnγ*: *P*_Linear_ = 0.280, *P*_Quadratic_ = 0.002.

The mRNA expression of genes related to pro-inflammatory cytokines including *tnfα* and *il1β* were significantly up-regulated when the FO in the diet was completely replaced by SO (*P* < 0.05) (**Figure 6**). With the increase of dietary OCT, the mRNA expression of *tnfα*, *ifnγ* and *il1β* showed a significant quadratic curve (*P* < 0.05), and the values in fish fed diets containing 0.7 and 2.1 g/kg OCT were lower than the SO diet. The expression of anti-inflammatory cytokines *il10* was up-regulated in a significantly quadratic pattern with the OCT increasing (*P* < 0.05).

## 4 Discussion

Dietary high SO levels might cause hepatic fat deposition in aquatic animals (4, 16), which was also confirmed in the present study. Previous study showed that OCT could effectively decrease adiposity and regulate energy balance (28–30). Whereas, the effects of supplemental OCT on hepatic lipid metabolism, antioxidant capacity and inflammatory response in fish have not been explored in detail. In this study, Oil Red O staining results showed that OCT supplementation could decrease the area of LDs induced by the SO diet. Meanwhile, the addition of OCT decreased the hepatic crude lipid, TG, TC, and serum TG and TC contents. The above results suggested that OCT can reduce the accumulation of lipids in the liver. We further explored how OCT affects hepatic lipid metabolism. Compared with the SO diet, the serum LDL-c content was reduced, and the serum HDL-c content was elevated in fish fed the diet containing 2.1 g/kg OCT. LDL-c accumulates a large amount of cholesterol in the arteriae intima and is generally considered to be one of the factors causing atherosclerosis. On the contrary, HDL-c could resist the accumulation, preservation and oxidation of LDL-c, and clear cholesterol back to hepatic from peripheral tissues (31). Similar results were observed in apoE^−/−^ mice, where the addition of OCT reduced the transport of cholesterol to extrahepatic tissues (32). Furthermore, NEFA is the main source of hepatic TG pool, released from adipose tissue by lipolysis, and high concentrations of NEFA can induce muscle and liver insulin resistance and induce dyslipidemia (33). NEFA concentrations can be reduced when fish were fed the diet containing 2.1 g/kg OCT. Consequently, these results demonstrated that OCT could ameliorate hepatic fat deposition and improve dyslipidemia induced by high-level SO diets.

To further elucidate the mechanism whereby OCT reduces the content of hepatic lipids, the mRNA expression of genes related to lipid metabolism was examined. In this study, the mRNA expression of transcription factors (*srebp1* and *cebpa*), genes related to *de novo* synthesis of fatty acids (*acc1*, *scd1*, and *fas*) and lipid synthesis (*dagt1*) were down-regulated in fish fed diets with OCT. Transcription factors (*srebp1* and *cebpa*) are essential for the coordinated regulation of lipid synthesis, regulating normal adipocyte differentiation and targeting molecules such as stearoyl-CoA desaturase-1 (SCD-1), acetyl-CoA carboxylase (ACC) and fatty acid synthetase (FAS) (34). Similarly, a reduction in the activity of DGAT2 and ACC mediated by OCT was observed in 3T3-L1 cells and human adipocytes (28). The down-regulation of these genes and proteins might be related to OCT-mediated PPARγ inactivation (35). With the addition of OCT, the mRNA expression of these genes first decreased and then increased, showing a significant quadratic trend, indicating that suitable concentration of OCT could alleviate lipid deposition, while excessive OCT may reverse this result, similar results were found in previous study (36). Besides affecting lipid synthesis, OCT also significantly altered the mRNA expression of FA transport-related genes. Compared with the FO diet, the mRNA expression of *cd36* in fish fed the SO diet was significantly up-regulated, and the addition of OCT reduced the expression of *cd36*. Similar results were found in H9c2 cells, OCT supplementation can restore the AVP (arginine vasopressin) induced down-regulation of *cd36* expression (37). VLDL assembly requires apoB-100 to mobilize triglycerides into the microsomal lumen (38). The supplementation of OCT also significantly up-regulated the mRNA expression of *apob100*, promoting hepatic VLDL assembly and secretion. Compared with the SO diet, the mRNA expression of genes involved in lipolysis (*atgl*, *hsl*, and *lpl*) and FA β-oxidation (*cpt1*, *aco* and *mcad*) were up-regulated in fish fed diets containing 0.7 and 2.1 g/kg OCT. MCAD is a key enzyme in the first step of the β-oxidation process of MCFA (39). A previous study also found that OCT significantly increased lipid oxidation-related gene expression and the protein expression of MCAD in AML12 cells (40). The possible reason is that compared with long-chain fatty acids, MCFA can enter the mitochondria independently of the carnitine transport system and be oxidized preferentially, which is crucial for the oxidative decomposition of FAs (41). Collectively, these results suggested that OCT could reduce the *de novo* synthesis of FAs, regulate the expression of FA transport-related genes, and promote lipolysis and FA β-oxidation thereby alleviating hepatic lipid deposition.

Lipid metabolism disorders may increase the production of reactive oxygen species (42). The imbalance of oxidative and antioxidant systems induces oxidative stress. T-AOC is the total antioxidant capacity, an effective marker of oxidative stress (43). Compared with the FO diet, T-AOC was significantly reduced in fish fed the SO diet, while it was improved with OCT supplementation. POD, SOD and CAT are key antioxidant enzymes to eliminate ROS accumulation (43). In this study, OCT treatment improved the antioxidant enzymes activity, which is consistent with studies in rats and AML12 cells (44–46). Excessive oxidative products damage biological molecules such as proteins, nucleic acids and lipids, impair their biological function, and trigger hepatic injury. MDA is the product of intracellular lipid peroxidation, and 8-OHDG is a marker of DNA oxidative damage (47). Hepatic MDA and 8-OHDG contents were decreased in fish fed with OCT. In summary, our results revealed the protective effect of OCT on hepatic oxidative stress induced by high SO levels diet.

Hepatic lipid accumulation may also lead to hepatocyte injury and inflammation (48). Supplementation of 2.1 g/kg OCT improved hepatocyte swelling and nuclear migration, and decreased serum ALT and AST levels, which are important biomarkers of hepatic injury. With the addition of OCT, Serum ALT and AST contents increased in fish fed the diet containing OCT 6.3 g/kg, and decreased in fish fed the diet containing OCT 18.9 g/kg, but there was no significant difference between them (*P* > 0.05). This result is consistent with subsequent inflammation-related genes (*tnf-α*, *il-1β* and *ifnγ*) expression, suggesting that liver damage might be mitigated by reduced inflammatory response. A previous study also found OCT prevented LPS-induced acute liver injury in rats (49). IL-1β, TNF-α and IFNγ are inflammatory cytokines, which initiate the production of a series of inflammatory mediators and produce inflammatory responses (48). In this study, the mRNA expression of *tnf-α* and *il-1β* were significantly up-regulated when the FO in the diet was completely replaced by SO, causing the occurrence of hepatic inflammatory response. After OCT intervention, the expression of *il-1β, ifnγ* and *tnf-α* were down-regulated, indicating that OCT could play an anti-inflammatory role, which is consistent with the results in mammals (50, 51). These results demonstrated that OCT could ameliorate hepatic injury and inflammation induced by high SO diets.

In conclusion, a concentration range of 0.7 and 2.1 g/kg OCT can significantly reduce hepatic lipid accumulation, relieve hepatic oxidative stress and regulate inflammatory response in large yellow croaker fed with high SO level diets. Collectively, OCT supplementation might have the potential to alleviate lipid metabolism disorders and hepatic damage, and provide a new way to improve hepatic lipid deposition in fish.

## Data availability statement

The original contributions presented in the study are included in the article/**Supplementary Material**. Further inquiries can be directed to the corresponding author.

## Ethics statement

The animal study was reviewed and approved by The Animal Experiment Ethics Committee of Ocean University of China and the Management Rule of Laboratory Animals (Chinese Order No. 676 of the State Council, revised 1 March 2017).

## Author contributions

MZ and QA designed the experiments, performed the main experiments and wrote the original draft. ZZ, YTL, and WZ conducted other experiments. MZ, YT, YG, and JZ analyzed the data. FC, GL, HZ, YRL, and KM revised the manuscript. All authors contributed to the article and approved the submitted version.
